# The gut microbiome, resistome, and mycobiome in preterm newborn infants and mouse pups: lack of lasting effects by antimicrobial therapy or probiotic prophylaxis

**DOI:** 10.1186/s13099-024-00616-w

**Published:** 2024-05-12

**Authors:** Elizabeth Y. Yuu, Christoph Bührer, Tim Eckmanns, Marcus Fulde, Michaela Herz, Oliver Kurzai, Christin Lindstedt, Gianni Panagiotou, Vitor C. Piro, Aleksandar Radonic, Bernhard Y. Renard, Annicka Reuss, Sara Leal Siliceo, Nadja Thielemann, Andrea Thürmer, Kira van Vorst, Lothar H. Wieler, Sebastian Haller

**Affiliations:** 1 Data Analytics & Computational Statistics, Hasso Plattner Institute, University of Potsdam, Prof.-Dr.-Helmert-Straße 2–3, 14482 Potsdam, Germany; 2 Charité - Universitätsmedizin, Berlin, Germany; 3 Robert Koch Institute, Berlin, Germany; 4 Department of Mathematics and Computer Science, Freie Universität Berlin, 14195 Berlin, Germany; 5 Institute for Hygiene and Microbiology, University of Würzburg, Würzburg, Germany; 6 Department of Microbiome Dynamics, Leibniz Institute for Natural Product Research and Infection Biology – Hans Knöll Institute, Beutenbergstraße 11A, 07745 Jena, Germany; 7 Faculty of Biological Sciences, Friedrich Schiller University, 07745 Jena, Germany; 8 Department of Medicine, The University of Hong Kong, Hong Kong, China; 9 Ministry of Justice and Health, Schleswig-Holstein, Kiel , Germany

**Keywords:** Preterm infants, Antibiotics, Infloran, Mice model, Microbiome, Resistome, Mycobiome

## Abstract

**Background:**

Enhancing our understanding of the underlying influences of medical interventions on the microbiome, resistome and mycobiome of preterm born infants holds significant potential for advancing infection prevention and treatment strategies. We conducted a prospective quasi-intervention study to better understand how antibiotics, and probiotics, and other medical factors influence the gut development of preterm infants. A controlled neonatal mice model was conducted in parallel, designed to closely reflect and predict exposures. Preterm infants and neonatal mice were stratified into four groups: antibiotics only, probiotics only, antibiotics followed by probiotics, and none of these interventions. Stool samples from both preterm infants and neonatal mice were collected at varying time points and analyzed by 16 S rRNA amplicon sequencing, ITS amplicon sequencing and whole genome shotgun sequencing.

**Results:**

The human infant microbiomes showed an unexpectedly high degree of heterogeneity. Little impact from medical exposure (antibiotics/probiotics) was observed on the strain patterns, however, *Bifidobacterium bifidum* was found more abundant after exposure to probiotics, regardless of prior antibiotic administration. Twenty-seven antibiotic resistant genes were identified in the resistome. High intra-variability was evident within the different treatment groups. Lastly, we found significant effects of antibiotics and probiotics on the mycobiome but not on the microbiome and resistome of preterm infants.

**Conclusions:**

Although our analyses showed transient effects, these results provide positive motivation to continue the research on the effects of medical interventions on the microbiome, resistome and mycobiome of preterm infants.

**Supplementary Information:**

The online version contains supplementary material available at 10.1186/s13099-024-00616-w.

## Introduction

Newborn infants are typically born with a sterile gut that rapidly colonizes with various microbes [44]. Particularly in preterm infants, gut-borne pathogenic microbes are capable of causing serious infections or even death [63, 66, 76]. Due to potential pathogenic bacteria exposure found in neonatal intensive care units, (NICUs), preterm infants are treated with systemic antibiotics for presumed or proven bacterial or fungal infection [6, 8, 22, 29, 32, 37, 77, 84]. The majority of very low birth weight infants receives antibiotics during the first days of life [34]. Antibiotic use in vulnerable patient populations is associated with complications and increases the risk of infection with multi-drug resistant microorganisms, thus antibiotic stewardship efforts have been increased [26, 34]. In addition to their heightened susceptibility to sepsis, preterm infants are vulnerable to rapid spreading acute transmural infections of the gut, clinically denoted as necrotizing enterocolitis (NEC) [4, 12, 32, 41, 45, 52, 54, 68, 75]. Although the use of empirical administration of antibiotics aids in reducing pathogenic risks, it also increases the risk of NEC [3, 21]. In contrast, the risk of NEC is decreased by prophylactic use of multiple-strain probiotics [14, 16, 23]. Nonetheless, these are simply two examples of how antibiotic and probiotic treatments may have an effect on the microbial population (microbiome) of preterm infants. In a recent review, similar studies discussed the impact of administering antibiotics on the gut microbiome [56]. While some studies reported a decrease in the abundance of beneficial phyla [28, 47, 62], other studies reported that administering antibiotics in combination with probiotics rebalanced and enriched the bacterial gut microbiome compositions [2, 5, 7, 19, 27, 31, 89]. Notably, other studies reported that regardless of which medical intervention preterm infants receive, the development of the gut microbiome will reach similar composition compared to the gut of an untreated full-term infant around the age of two [50, 59]. Apart from the microbiome, a second key component of the gut is the resistome, the composition of antibiotic resistance genes (ARG)s [31, 78]. Similar to the gut microbiome, it remains unclear how antibiotic therapy influences the resistome development in preterm infants. Previous research have demonstrated that certain antibiotic treatments promote the cultivation of ARGs which then can contribute to an increased growth of pathogens [31, 78]. A third key component of the gut is the mycobiome, the fungal community. Although the mycobiome amounts to a small portion of the human gut, it may also affect infant health, for example, harboring pathogenic fungi [20]. Some studies suggest that age is an influencing factor such that infants have more diverse and richer compositions compared to adults [36], while other studies suggest the contrary [80].

To expand our understanding of how medical interventions and other potential factors (ie. birth mode, nutrition, and multiple births) impact preterm infants’ gut compositions, we conducted a prospective quasi-intervention study supported by a controlled neonatal mouse model.

## Materials and methods

Preterm infants were exposed to routine interventions according to age and clinical needs. A mouse model was designed to closely reflect exposures in humans. Preterm infants and neonatal mice were grouped into four groups: antibiotics only, probiotics only, antibiotics followed by probiotics, and no intervention/none. Within the antibiotics only category, eight different antibiotic combinations were administered to the preterm infants and two different antibiotic combinations were administered to the mice. Stool samples from both the preterm infants and mice were collected at varying time points.

### Preterm infants

Our study included preterm infants from two NICUs from the Charité Mitte and the Charité Virchow hospitals in Berlin, Germany (August 2018-June 2019). Written consent was obtained from the parents or infant guardians. The inclusion criteria for the infants were as follows.

**Study population**

- Born less than 37 weeks of gestational age
- Age 1–7 days of life
- Birth weight between 1250–1750 grams
- Hospitalized in the NICUs of either Charité hospitals

Treatment groups: the following antibiotics were administered according to the local standard procedures (Ampicillin/Gentamicin, Gentamicin/Meropenem, Unacid/Vancomycin, Ampicilli/Clindamycin, Unacid/Gentamicin/Vancomycin, Unacid/Gentamicin/Clarithromycin, Unacid/Gentamicin/Flucloxacin, and Ampicillin/Vancomycin/Meropenem). According to the standard protocol only infants with birth weight below 1500 grams or postmenstrual age below 32 weeks received a ten-day course of probiotics (Infloran^®^, consisting of 109 colony-forming units *Lactobacillus acidophilus* and 109 colony-forming units *Bifidobacterium infantis*.) For further information on medical interventions among the four categories of preterm infants, please refer to Additional file 1: Table S1.

**Fig. 1:**
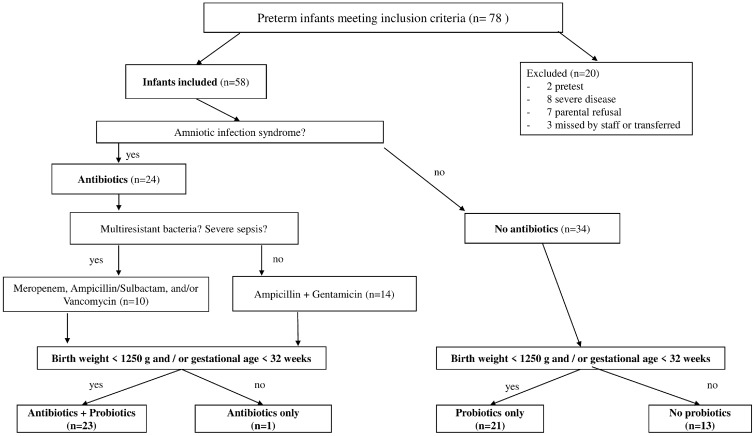
Overview of the preterm infants’ inclusion criteria and their allocation to the treatment groups. Of the 78 preterm infants, 58 were included in the study

Data and specimen collection: Using a standard questionnaire, we collected demographic and clinical information including sex, birth weight, gestational age, birth mode, birth place, nutrition, ventilation, type and timing of probiotic and antibiotic treatment. Sepsis was defined according to clinical appraisal. At least 3 stool samples were collected from each infant: between day 1 and 3, between day 9 and 17, and the final day prior to discharge (see Fig. 1) .

### Neonatal mice

Study population: Neonatal mice were locally bred and held under specific pathogen-free conditions (SPF) at the animal facility of Freie Universität Berlin.

Treatment groups: On days 6 and 7 of life, each neonatal mouse received either antibiotics or phosphate buffered saline (PBS). In addition, the neonatal mice were treated on day 6 with one of the following assignments: no antibiotics/no probiotics, no antibiotics/probiotics, Ampicillin/Gentamicin/no probiotics, Ampicillin/Gentamicin/probiotics, Meropenem/Vancomycin/no probiotics, Meropenem/Vancomycin/probiotics. Neonatal mice that were administered probiotics (Infloran®), received their treatment orally twice a day for 10 days (between days 8 and 17 of life). The probiotics treatment consisted of 1$\upmu$l suspensions of dissolved Infloran^®^ from sterile PBS with at least 2*106 colony forming units. Paired antibiotics of Ampicillin/Gentamicin, and Meropenem/Vancomycin were each given in doses of 150 mg/kg/day. For full medical intervention distribution, please refer to Additional file 2: Table S2. The neonatal mice were kept in treatment specific cages. All treatment groups were assigned litter-wise, such that all animals from one litter and one dam were consistently equally treated. To minimize external influences, whole litters were treated as one treatment group. The administration of antibiotic combinations was different between the groups: Ampicillin, Gentamycin and Vancomycin were applied orally, Meropenem was injected subcutaneously in the nuchal fold. We did not observe any procedural failure during our experiments. All animals were weaned by the age of 21 days.

Specimen collection: At least 2 fecal pellets were collected on day 18 and day 42 of life. These pellets were placed in sterile tubes and stored in an -$80^{\circ }C$ environment. The fecal pellets were transferred to the Robert Koch Institute and the Institute for Hygiene and Microbiology in Würzburg for analysis.

### Sample preparation and sequencing

#### Microbiome and resistome

QIAamp Fast DNA Stool Mini kits (Hilden, Germany), were used for genomic DNA extraction from stool samples. The Illumina MiSeq platform was used to sequence the 16S rRNA on the V3–V4 regions and Illumina HiSeq was used for shotgun metagenomics sequencing.

#### Mycobiome

All available stool samples of the preterm infants and the neonatal mice were processed by LGC Genomics Berlin, Germany. The DNA was isolated with the Qiagen DNeasy PowerSoil Kit (Hilden, Germany) with four parallel DNA extractions per sample to obtain sufficient fungal DNA. The DNA from parallel extractions was pooled together for sequencing. The Illumina Miseq V3 with paired-end reads of 300bp (2x150bp) was used for all samples. The Internal Transcribed Spacer 1 (ITS1) region was amplified using ITS1F/ITS2R primers.

### Bioinformatics

Two different approaches were performed for the abundance and diversity analyses, one for the microbiome and the resistome, and one for the mycobiome. Analyses focused on various taxa levels: phyla, family, genera, and species.

#### Microbiome and resistome analyses

All microbiome and resistome analyses were performed using the command-line versions of the metagenomics tools listed below. Full description of the specific parameters can be found in the supplement. All commands were executed locally on a laptop.

Preprocessing: For the 16S rRNA data, we used the QIIME2 command line platform version 2021.4 with default parameters for quality control, filtering, chimera detection, de-noising, detection of sequence variants and taxonomic classification [9, 15]. For the WGS data, we used command lines platforms of the following tools with default parameters, Fastp version 0.20.0 for quality control, Bowtie2 version 2.3.5, and bedtools version 2.29.2 for removal of host sequences [18, 48, 70].

Classification: To compare our datasets on different levels of resolution, we analyzed the 16S rRNA and WGS datasets using three different bioinformatics tools: QIIME2 version 2021.4 (lower resolution), MetaPhlAn2 version 2.9.21(lower resolution) and Ganon version 0.2.2 (higher resolution) [9, 67, 83]. Taxonomic classification on the 16S rRNA datasets was performed using QIIME2 with default parameters against the SILVA database [9, 69]. Taxonomic classification on the WGS datasets was performed using MetaPhlAn2 and Ganon.

Antibiotic resistance genes: For the antibiotic resistance analysis, we used the command line platforms of the tools groot version 1.0.2 and srst2 version 0.2.0 against the resistance gene identifiers in the Comprehensive Antibiotic Resistance Database (CARD), ResFinder, and the Antibiotic Resistance Gene Annotation (ARG-ANNOT) databases [33, 38, 55, 72, 90].

Abundance comparisons: QIIME2 was used for the 16S rRNA dataset, while internal scripts based on the scikitbio library were used for the WGS dataset. From the three classification tools, we generated three count tables. We performed an analysis of compositions of microbiomes (ANCOM) on each count table to test for differential abundance [51]. We concluded significant differences based on the W statistics produced from the ANCOM [51]. Visuals for abundance related figures were generated using the command line platform of GRIMER version 1.0.0 [67]. Conclusions and inferences on the relative abundances were drawn from GRIMER outputs.

#### Mycobiome analysis

Processing: We used the PIPITS pipeline version 2.4 for taxonomy annotation of fungal ITS with default parameters that included quality filtering, read-pair merging, ITS1 extraction and chimera removal [35]. We binned the remaining reads based on 97% similarity as operational taxonomic units and aligned QIIME2 to the UNITE fungi database using mothur [9, 46, 74]. We then normalized the preterm infant and neonatal mice samples by cumulative sum scaling using R package metagenomeSeq [9, 64].

Diversity analysis: Alpha diversity indices detailing mycobiome community composition within samples were calculated using the R packages vegan [24]. Testing for significant differences in alpha diversity was performed using Wilcoxon signed-rank test. For estimating beta diversity reflecting community dissimilarities, Bray-Curtis distances were calculated using the R package vegan [24]. To test for significant differences within the mycobiome composition, beta diversity was calculated using permutational multivariate analysis of variance (PERMANOVA) as implemented in the function Adonis from the R package vegan [24].

Abundance comparisons: For the neonatal mice samples, differentially abundant features were identified by the Wilcoxon rank-sum test and were considered significantly different in abundance if the p-value was less than 0.05. For human samples, differentially abundant features were identified by generalized linear mixed model using the R package glmmTMB adjusting for birthplace, multiple birth, time period and birth mode, and nutrition. Information about the use of formula or mother milk, pre-nutrition and/or supplements was considered when referring to the nutrition. Features were considered significantly differentially abundant if the p-value was less than 0.05 [11].

## Results

We collected 185 samples from 58 preterm infants for the microbiome and resistome analyses. Sequencing for these analyses resulted in one 16S rRNA dataset and one WGS dataset. The 16S rRNA dataset had an average of 57,000 paired-end reads of 600bp (2 × 300 bp) and the WGS dataset had an average of 6.8 million paired-end of 250bp (2 × 125 bp) reads per infant sample. For the mycobiome analysis of 52 samples from 17 preterm infants sufficient material was available. The average total read count was 53,000 reads per infant sample. Of the 48 neonatal mice, we collected and sequenced 70 samples for microbiome and resistome analyses. The 16S rRNA dataset and WGS dataset had an average of 93,000 paired-end reads of 600 bp (2 × 300bp) and of 4.5 million paired-end reads of 250 bp (2 × 125bp) per mouse, respectively. For the mycobiome analysis, we collected and sequenced 61 samples that resulted in an average total read count of and 47,000 reads per mouse sample (see Table 1).

**Table 1:** Demographic, clinical characteristics and treatment of preterm infants (n = 58)

Preterm neonates (n=58)		Number (% among all 58 neonates)
Sex	Male	37 (64%)
	Female	21 (36%)
Birth weight (g)	Median (interquartile range)	1490 (1255–1750)
Birth mode	Vaginally delivered	10 (17%)
	Primary cesarean section	13 (23%)
	Secondary cesarean section	35 (60%)
Ventilation	No	8 (14%)
	Nasopharyngeal	44 (76%)
	Nasopharyngeal/intratracheal	6 (10%)
	Intubated	24 (41%)
Clinical sepsis	Early onset	19 (33%)
	Late onset	6 (10%)
Bacterial colonisation	Positive rectal screening for resistant bacteria	8 (14%)
Probiotics	One course	44 (76%)
	Two courses	4 (7%)
Antibiotics	During pregnancy	18 (31%)
	During birth	54 (93%)
	During breast feeding	14 (24%)
	After birth (<72 hours)	19 (33%)
	After birth (>72 hours)	6 (10%)
	Ampicillin/Gentamicin (after birth)	14 (24%)
	Other antibiotics (or antibiotics combinations after birth)	10 (17%)
No antibiotics		34 (59%)

The numbers of exposures do not necessarily add up to 100% due to multiple exposures

### Microbiome

**Fig. 2:**
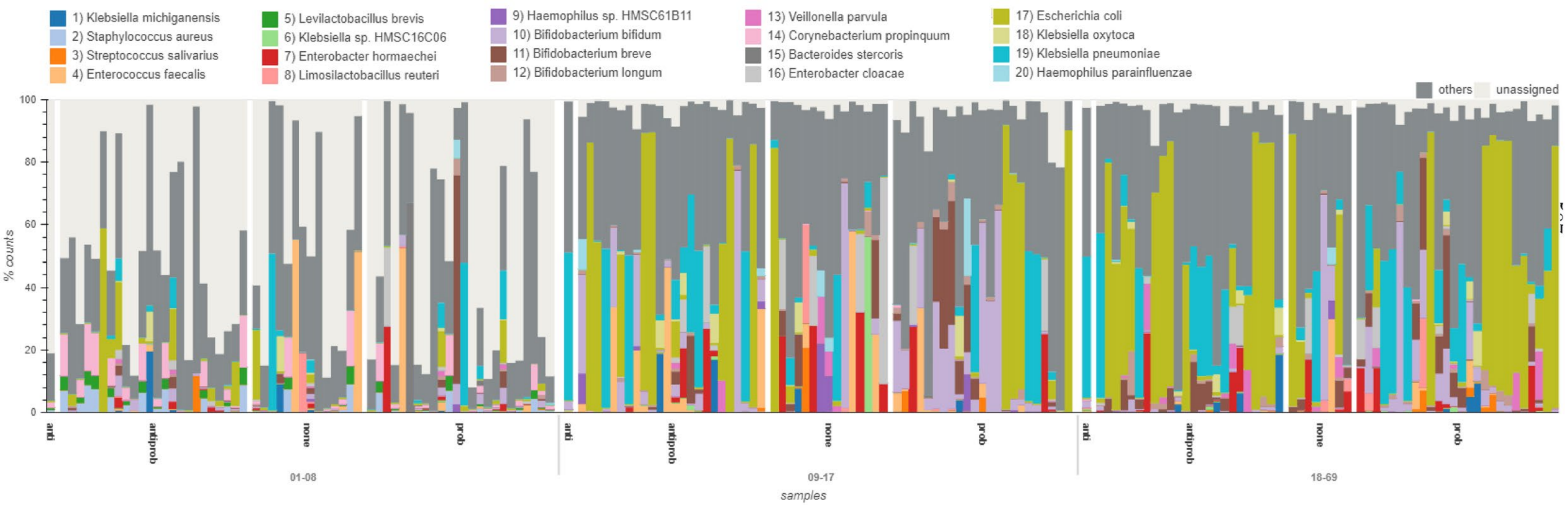
Microbiome composition at species level in preterm infants analyzed using the WGS dataset, classified with Ganon and adapted from Grimer. Data were stratified by treatment group and time points. The x-axis displays three time points (days 01–08, 09–17, and 18–69) and treatment groups (anti, antibiotics only; antiprob, antibiotics with probiotics; none, no treatment; and prob, probiotics only). Each bar represents an individual sample, and the y-axis indicates the percentage of reads mapped to specific taxa

Throughout the duration of the study, an increase in microbiome richness was observed across all treatment groups. Although individual samples within the treatment groups exhibited variability, no significant differences were detected between treatment groups (see Fig. 2).

ANCOM analyses were conducted on both 16S rRNA and WGS datasets using the three classification tools: QIIME2, MataPhlAn2, and Ganon. At the second time point, the species *Bifidobacterium bifidum* was found differentially abundant (W statistics QIIME2, MataPhlAn2, and Ganon: 61, 111, 249) in each treatment group compared to the other species detected. After stratifying the data based on delivery mode, additional species were found differentially abundant, but not in both 16S rRNA and WGS datasets or from all bioinformatics methods. Detailed findings of these specific results are presented in the Supplement. In the following abundance results, we report our observations based on the visuals produced using GRIMER.

One infant was allocated in the antibiotic only group, Ampicillin/Gentamicin. The dominant species for this infant was *Klebsiella pneumoniae* at the second and third time points. The infants that received no treatment/ PBS oral, presented no patterns nor similar prominent taxa at any of the time points. In the probiotics only group, we observed the following three prominent species at the second and third time points: *Bifidibacterium bifidum*, *Escherichia coli*, and *Klebsiella pneumoniae*. In the antibiotics with probiotics group, we observed abundance patterns regardless of which antibiotics administered. Out of the 23 infants in the antibiotics with probiotics group, 10 infants showed a strong presence of *Escherichia coli* at the second and third time points. We observed no traces of *Escherichia coli* at any time point in the Ampicillin/Vancomycin/Meropenem and Unacid/Gentamicin/Vancomycin groups. In addition and regardless of antibiotics combination, we observed 7 infants that had *Klebsiella pneumoniae* as the dominant species in the third time point. Two infants, 126 and 112, with the following antibiotics combination, Unacid/Gentamicin/Flucloxacin and Gentamicin/Meropenem, respectively, had traces of *Escherichia coli* only at the first time point. After the first time point, we no longer detect *Escherichia coli*.

Triplet, twins and singletons: In our study population, we analyzed the subset of twins (7 pairs; 14 infants) and triplets (1 set; 3 infants). See Additional file 4: Table S4 in the Supplement for full multiple births distribution. Multiple birth sets, comprising of infants born from the same womb, enabled the study to examine the development of gut compositions that originated from a shared environment. Within this cohort, certain sets received identical treatments while others did not.

**Fig. 3:**
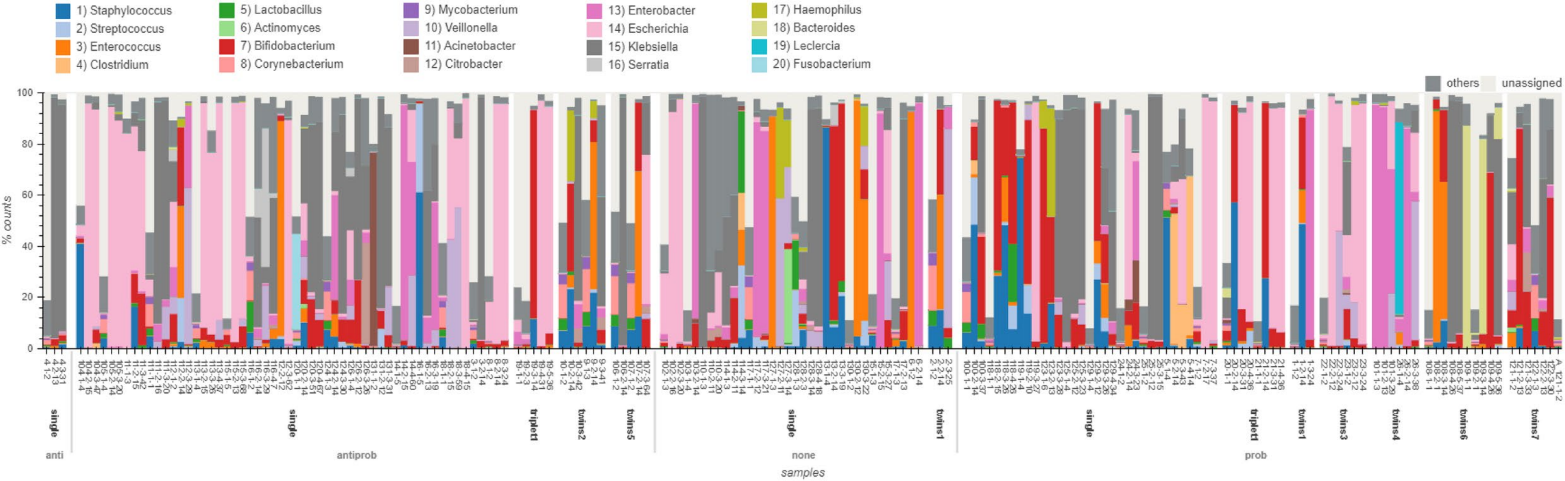
Microbiome composition at genus level in preterm infants analyzed using the WGS, classified with Ganon,and adapted from Grimer. Data were stratified by birth type (triplets, twins, or singletons) and treatment group. The x-axis displays treatment groups (anti, antibiotics only; antiprob, antibiotics with probiotics; none, no treatment; and prob, probiotics only), birth type and additional information (sample id-sample number-sampling day). Each bar represents an individual sample, and the y-axis indicates the percentage of reads mapped to specific taxa

Among the triplets, two individuals received probiotics only while one received antibiotics (Ampicillin/Gentamicin) followed by probiotics. Despite the different treatments, the genera abundances within the triplets exhibited similar trends. At the second time point, *Staphylococcus* and *Bifidobacterium* were the dominant taxa in the triplets. Subsequently, at the final time point, *Escherichia* emerged as the dominant taxon in the triplets. Twins 106 and 107 both received probiotics but different antibiotic combinations. Infant 106 received Unacid/Gentamicin/Vancomycin and infant 107 received Unacid/Vancomycin. The last time point for subject 106 was dominated by *Klebsiella* whereas infant 107 was dominated with *Escherichia*. Another set of infants that did not receive the same treatment were infants 1 and 2. Infant 1 received probiotics only and infant 2 received no treatment. For the second time point, both infants presented similar compositions, *Bifidobacterium*, *Enterococcus* and *Staphylococcus*. However, at the third time point, day 24 for infant 1 and day 25 for infant 2, the compositions diverged. Infant 2’s composition was mostly comprised of *Veillonella* whereas infant 1’s composition was mostly *Enterobacter*. Of the 7 pairs of twins, 4 pairs (8 infants) were administered probiotics only. The individual pairs presented very similar taxa abundances within their own pairing, however, each pair had distinct patterns. Twins 22 and 23, received probiotics only and were both *Escherichia* dominant at all time points. Twins 26 and 101 were *Enterobacter* and *Veillonella* dominant. Twins 108 and 109 were found *Bacteroides*, *Bifidobacterium*, and *Klebsiella* dominant. Lastly, twins 121 and 122 were *Bifidobacterium* and *Klebsiella* dominant. See Fig. 3 for additional information on the multiple births.

Pathogens and infections: Among the preterm infants, 19 individuals developed early-onset sepsis (EOS, within the first 72 h of life), and 6 other individuals developed late-onset sepsis (LOS, later than 72 h of life). We observed no differences in abundance or composition patterns when comparing infants with EOS or LOS compared to those without either condition. All sample compositions, with or without EOS/LOS, showed similarity in the number of genera identified and were mostly comprised of *Klebsiella*, *Escherichia* and *Enterobacter*. To identify potential colonization of multiresistant bacteria, weekly rectal screenings were performed. We detected two prominent pathogen species: *Staphylococcus aureus* and *Escherichia coli*. However, infants with these species had similar microbial compositions as to those without these species. No distinct patterns were observed in the microbiome compositions when comparing EOS/LOS infants to infants who tested positive for pathogenic species during screening.

#### Resistome

Using the ARG-ANNOT database with a sequence similarity search cutoff of 97%, we identified 27 antibiotic resistant genes (ARGs), 7 of which were encoded by plasmids and are the ones we report. The greatest percentages of ARGs were found in the antibiotics with probiotics group (*bla* gene: 41.67%), and probiotics only (*Sul*, *Tet* and *Tmt* genes: 26.92%, 23.08%, and 30.43%) groups. In the other treatment groups, other ARG genes had lower percentages ranging from 0% to 4%. See Additional file 3: Table S3 for further ARGs information. The greatest percentages of ARGs were found in the antibiotics with probiotics, and probiotics only groups. We observed a notable increase regarding genes conferring resistance to beta-lactams (*bla*) antibiotics. Specifically, at the initial time point, approximately 12.5% of the samples showed signs of *bla* genes present in the antibiotics with probiotics group which then increased to 41.67% by time point three. The data, classified from Ganon, also presented a shift in taxa abundances between the first and third time points. At the first time point, the gut compositions consisted of *Staphylococcus*, *Klebsiella*, and *Enterobacter*. However, at the third time point, the gut compositions were dominated by the genera *Bifidobacterium*, *Enterobacter*, *Escherichia*, *Klebsiella* and *Veillonella*. Another noticeable shift in abundance between time points occurred in the probiotics only group. At the first and second time points in the probiotics only group, there was no detection of the resistance genes against Sulfonamide (*Sul*) and Tetracycline (*Tet*). At the third time point, the percentages of *Sul* and *Tet* increased to 26.92% and 23.08% respectively. The most prominent genus at the first time point was *Klebsiella*, whereas, at the third time point, the gut was dominated by *Klebsiella*, *Escherichia*, and *Bifidobacterium*.

#### Mycobiome

**Fig. 4:**
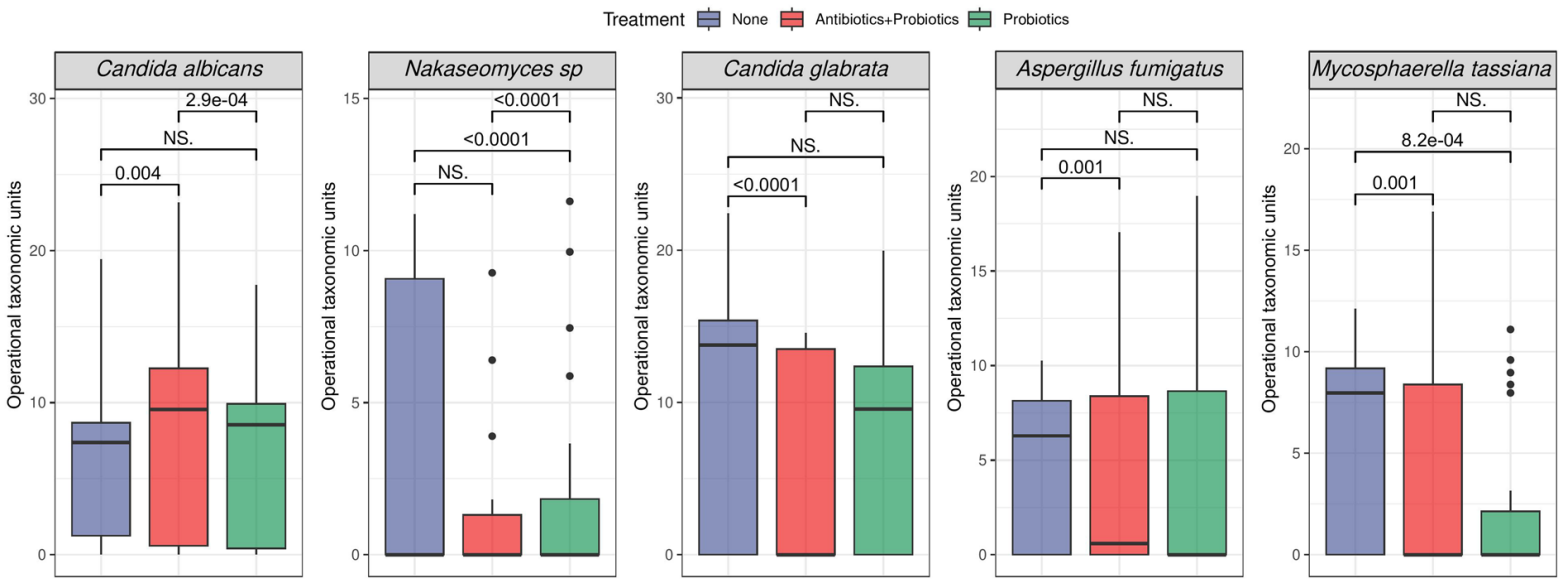
Figure of abundance of fungal species in preterm infants by treatment group (None: no treatment, antibiotics with probiotics and probiotics only) produced from R. Statistical significance between groups was determined using generalized linear mixed models. NS: not significant

There were no samples available for the antibiotics only group, therefore, we only performed the mycobiome analysis using three treatment groups (no treatment, antibiotics with probiotics and probiotics only). We observed high intra-variability in the different treatment groups at the genus level, however, these differences were not significant in alpha diversity using Shannon and Simpson index (Wilcoxon rank-sum test, p-values greater than 0.05). The differences in mycobiome structure between the treatment groups were also not significant in beta diversity using the Bray Curtis distance. We found no significant changes in alpha and beta diversity by birth mode. Birth place and multiple births had significant impact on alpha diversity at the genus level (birth place: p-values = 0.0012 and 0.001 for Shannon and Simpson respectively, multiple births: p-values = 0.0084 and 0.0057 for Shannon and Simpson respectively; alpha = 0.05). We observed that twins had lower alpha diversity compared to singletons. Beta diversity at genus level between birth places was also found significantly different (PERMANOVA, p-value- = 0.04, alpha = 0.05). We then used a generalized linear mixed model (GLMM) approach, adjusting for birthplace, number of births, birth mode,time, and nutrition to find species with significantly different abundance between the no treatment, antibiotics with probiotics and probiotics only groups. We found 5 species that had significantly different abundances between the treatment groups: *Candida albicans*, *Nakaseomyces sp*, *Candida glabrata*, *Aspergillus fumigatus *, and *Mycosphaerella tassiana* (see Fig. 4).

### Neonatal mice

Of the 48 neonatal mice, we collected and sequenced 70 samples for microbiome and resistome analyses. The 16S rRNA dataset and WGS dataset had an average of 93,000 paired-end reads of 600bp (2 × 300 bp) and of 4.5 million paired-end reads of 250bp (2 × 125 bp) per mouse, respectively. For the mycobiome analysis, we collected and sequenced 61 samples that resulted in an average total read count of and 47,000 reads per mouse sample

#### Microbiome

**Fig. 5:**
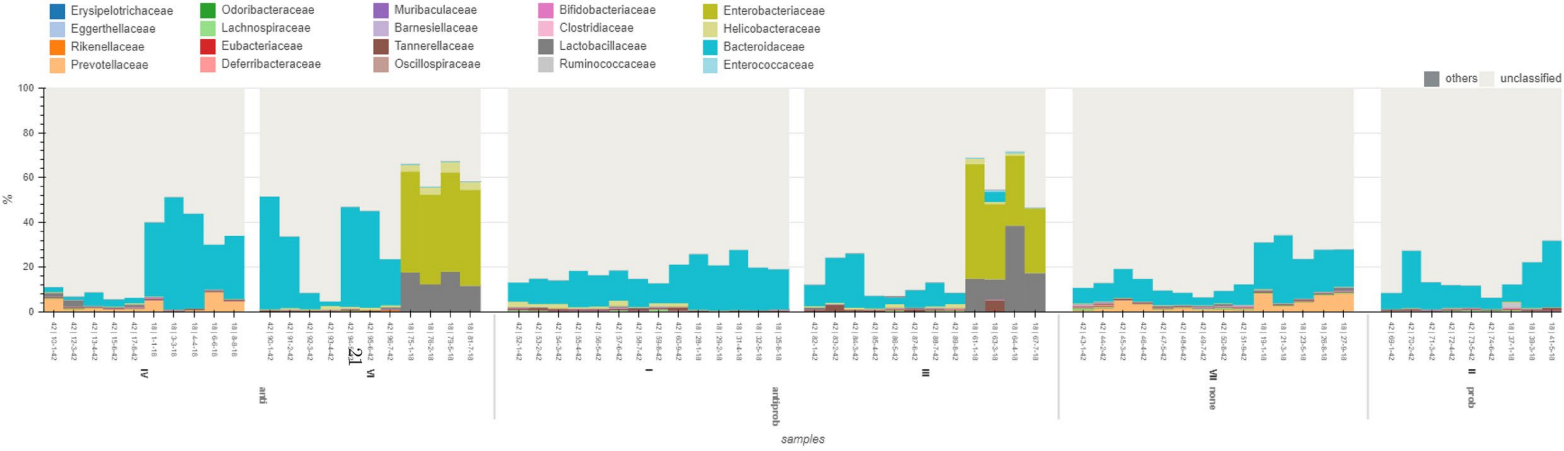
Microbiome composition at family level in neonatal mice analyzed using the WGS dataset, classified with Ganon and adapted from Grimer. The data were stratified by treatment group and time points (Stratum V is missing due to mislabeling during the wet lab process). The x-axis displays treatment groups (anti, antibiotics only; antiprob, antibiotics with probiotics; none, no treatment; and prob, probiotics only), specific antibiotic exposure (I = Ampicillin/Gentamicin + Infloran, II = Infloran, III = Meropenem/Vancomycin+ Infloran, IV = Ampicillin/Gentamicin, VI = Meropenem /Vancomycin, VII = None (PBS oral)), and additional information (sampling day | sample ID - sample number - sampling day). Each bar corresponds to an individual sample and the y-axis indicates the percentage of reads that mapped specified taxa

To test for significant differences in taxa abundance, we performed an Analysis of Composition of Microbiomes (ANCOM) on the neonatal mice data classified from three tools: QIIME2, Ganon, and MetaPhlAn2. Unlike the preterm infant data, the ANCOM results from the three classified datasets, did not produce similar statistical conclusions. Therefore, we report the statistical results from the tool that provided the highest taxonomic resolution, Ganon. Individual neonatal mice in the same treatment group demonstrated similar microbiome compositions and different treatments corresponded to specific changes in the microbiome compositions. Overall, *Bacteroidaceae* was noticeably the dominant family in treatment groups I, II and VII at both time points. *Bacteroidaceae* was only undetectable at the first time point in groups III and VI. Using ANCOM, *Bacteroidaceae* (W statistic 7,) and *Muribaculaceae* (W statistic 7) were found differentially abundant at the first time point and in all the treatment groups except for groups III ( Meropenem/Vancomycin + Infloran) and VI ( Meropenem/Vancomycin). Although *Muribaculaceae* was found differentially abundant, it was not noticeably observable by visualizing the data. In contrast, *Enterobacteriaceae*, *Heliocobacteraceae* and *Lactobacillaceae* (W statistics: 7, 8,7 ) were present in groups III ( Meropenem/Vancomycin + Infloran) and VI (Meropenem/Vancomycin). These three families were absent if not exhibited minimal detectability at the second time point (W statistics: 0, 0, 7). We observed a presence of the family *Helicobacteraceae* at the second time point in group I (Ampicillin/Gentamicin + Infloran ) and no detection of this taxon in group IV (Ampicillin/Gentamicin). This family was not differentially abundant in either treatment group. In contrast, we observed the family *Prevotellaceae* at both time points and in group IV; this family was found differentially abundant (W statistics: 8, 6). We did not observe any traces of *Prevotellaceae* in group I. *Lachnospiraceae* was found differentially abundant in groups II (probiotics only) and VII (no treatment), *Porphyromonadaceae* was differentially abundant in groups II, IV and VII for time point one, and groups IV and VI at time point two (W statistic 8), and *Tannerellaceae* was differentially abundant in groups II, III. To summarize, three bacterial families were found differentially abundant in both time points: *Lactobacillaceae*, *Porphyromonadaceae*, and *Prevotellaceae* on day 18 (W statistics: 7, 8, 8) and day 42 (W statistics: 7, 8, 6). *Prevotellaceae* remained consistently differentially abundant in treatment groups IV and VII (see Fig. 5).

### Resistome

Using the ARG-ANNOT database, we identified one antibiotic resistance gene from a plasmid and only at the first time point in the Meropenem/Vancomycin treatment group: Bla ACT-5. This gene was present in 50% of samples with a sequence similarity search cutoff of 97%. At the first time point, the gut was comprised of the families *Enterobacteriaceae* and *Lactobacillaceae*. At the second time point there were no traces of the previous families, instead, there was only one prominent family, *Bacteroidaceae*.

### Mycobiome

**Fig. 6:**
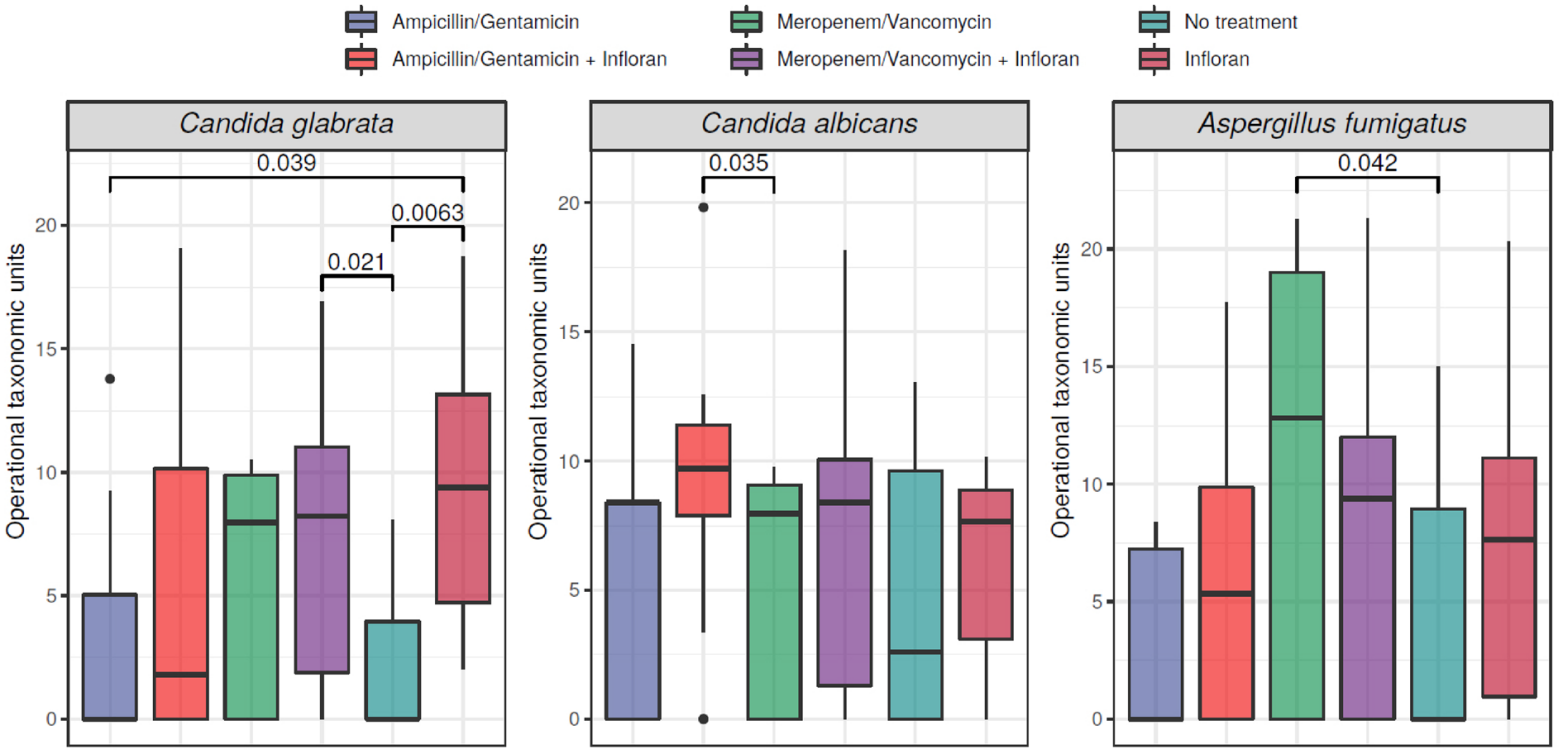
Boxplots of the fungal species abundances of the neonatal mice by the different treatment groups produced from R. Infloran: probiotic treatment. Statistical significance between groups was determined using the Wilcoxon rank sum test

Application of the Wilcox rank-sum test revealed three species significantly different in abundance: *Candida glabrata*, *Candida albicans*, and *Aspergillus fumigatus*. All three of these species presented results indicating statistically significant increases in abundance. The statistically significant increase in abundance of *C. glabrata* was observed when comparing the probiotics only group to three other treatment groups respectively, no treatment, Ampicillin/Gentamicin and Meropenem/Vancomycin with Infloran^®^ (p-values: 0.0063, 0.039,0.021, alpha = 0.05). The statistically significant increase in abundance of *C. albicans* was observed regarding in the Ampicillin/Gentamicin with Infloran^®^ group compared to the Meropenem/Vancomycin group (Wilcoxon rank-sum test, p-value = − 0.035, alpha = 0.05). Finally, *Aspergillus fumigatus* significantly increased in abundance in the Meropenem/Vancomycin group compared to the no treatment group (Wilcoxon rank-sum test, p-value = − 0.035, alpha = 0.05) (see Fig. 6).

## Discussion

We conducted a prospective quasi-intervention study aimed to understand how administering antibiotics, and probiotics influence the gut microbiome, resistome and mycobiome of preterm infants supported by a controlled neonatal mice model. Our data did not detect long lasting effects but rather transient alterations in the bacterial and fungal communities.

### Probiotics effects and environmental factors on the preterm infants

From our microbiome analysis, we found *Bifidobacterium bifidum* differentially abundant compared to the other species detected in each treatment group and more prominent in the probiotics only, and antibiotics with probiotics groups. The W statistics, (W statistics: 61, 111, 249), and greater percentage of samples containing *Bifidobacterium bifidum* in the probiotics only and the antibiotics paired with probiotics groups would suggest that exposure to probiotics, with or without the addition of antibiotics, contributes to higher prevalence of *Bifidobacterium bifidum*.

Our research aligns with other works such that while the genus *Bifidobacterium* was observed in all treatment groups, it was most prominent in the probiotics treatment group [1, 27, 86, 88]. A recent review discussed the impact of administering antibiotics on the gut microbiome specifically in children between 0–12 years of age [56]. In this review, some studies reported a decrease in abundance of the gut beneficial genera *Bifidobacterium* and *Lactobacillus*, and an increase in the abundance of the pathogen-prone phylum *Proteobacteria* and genus *Enterococcus* [28, 47, 62]. Other studies reported that administering antibiotics in combination with probiotics rebalanced and enriched the bacterial microbiome gut compositions, allowing preterm infants to mature properly [2, 5, 7, 19, 27, 31, 89]. In addition, a study in Norway showed that preterm infants who received higher doses of antibiotics and probiotics developed higher relative abundances in *Bifidobacterium* and *Lactobacillus* compared to preterm infants who received lower doses of antibiotics and probiotics. They also saw significant increases in alpha diversity with regards to age in the probiotics and non-probiotics groups compared to the full-term infants. These similar works offer positive evidence that the combination of antibiotics and probiotics is beneficial for preterm infants [27]. In contrast, other studies reported that regardless of which medical intervention preterm infants receive, the development of the gut microbiome will reach similar composition compared to the gut of an untreated full-term infant around the age of two [50, 59].

In our study, we had a set of triplets and 7 pair of twins, and of these individuals, not all infants received similar treatments. Of the triplets, two received no treatment and one received Ampicillin/Gentamicin. Despite receiving different medical interventions, the samples indicated that all three individuals developed similar gut compositions. Moreover, all the samples were acquired on the same days and all three gut compositions followed the same abundance trends. On day 14, all three individuals had *Bifidobacterium* and *Staphylococcus* dominant samples. On days 31 and 36, the three compositions were fully if not mostly comprised of *Escherichia*. As mentioned in the results section, twins 106 and 107 received probiotics, however, different antibiotics combinations. Although the genera abundances indicated that the infants had two distinct gut compositions, and this may appear as a significant result, it should be noted that time may have been an influencing factor. The dissimilarities between the two compositions were noticeable, however, there were only two time point samples, on day 2 and day 14 for infant 106, whereas, infant 107 had an additional sample collected later in the study which totaled to 3 samples that were taken on days 2, 14 and 64. Finally, of the 7 pairs of twins, we administered the same treatment, probiotics only, to 4 pairs (8 infants). Although receiving the same treatment, the individual pairs presented distinct patterns from each other, but followed similar composition patterns within their own pairing.

These observations from the twins/triplets would suggest that environmental and genetic factors, such as host genetics (from the mother), skin contact from the parent/guardian and exposure to the same hospital environment, have strong influence on the gut development. Other works have also demonstrated that twins/triplets presented similar gut compositions compared to unrelated infants and also suggest that the environmental factor strongly influence the gut development [17, 79].

Another key component of the gut is the resistome, the composition of antibiotic resistance genes (ARG)s [31, 78]. We detected three resistance genes, Bla, Sul and Tet. Interestingly, we detected these ARGs the probiotics only, and the antibiotics with probiotics treatment groups. Previous works have shown that certain antibiotic treatments enrich the cultivation of ARGs which can then lead to increased pathogen development [31, 78]. Similar to other studies [27, 31], we detected more ARGs (especially *bla* genes) in preterm infants who were exposed to antibiotics only or antibiotics with probiotics. In contrast, genes conferring resistance to Sul and Tet, were found only in the probiotics only group. In a clinical setting, probiotics is administered to reduce pathogen prone taxa and increase beneficial taxa [73]. In our study, we found occurrences of antibiotic resistance genes in the probiotics treatment groups. Although, we could not find similar developments in other studies we suspect environmental or genetic factors contributed to these results. Another interesting perspective to consider would be that the probiotics, Infloran^®^, contributed to the development of increased abundance of pathogen prone taxa. Therefore, we would strongly suggest further research on the effects environmental and genetic factors have on medical interventions, in addition to further experiments administering Infloran^®^ on preterm infants to better understand its influence on gut development.

As for the mycobiome, our analysis revealed that birthplace and multiple births had significant impact on alpha and beta diversity. This analysis also aligned with other preterm infant studies. We found that *Candida* was the dominant genus in all treatment groups [39, 53] Studies have reported that because of vertical transmission between a mother and infant, *Candida* increases in abundance [85]. However, we found no significant differences in alpha and beta diversity between the different birth modes. We found significant differences in the mycobiome between singletons-multiple births and birth place. These findings strongly suggest that environmental and genetic factors may influence the gut mycobiome development.

### Probiotics effects in the mouse model

Our study included a murine model to deduce if the use of mice could predict medical intervention effects in preterm infants. Due to the nature of a mice study, we were able to include more subjects in the different treatment groups compared to the preterm infant groups that had fewer or no subjects. Despite having more subjects allocated in the different groups, there were limited samples in each treatment group. Therefore, we suspect that statistically significant findings were likely the result of low abundances. Regardless of the sample sizes, we reported our statistical findings in addition to the general changes in taxa. The microbiome compositions of the preterm infants and mice exhibited no similarities in taxa abundances. However, as seen in the preterm infants and mice, medical intervention influences were observed, but transient. For the mycobiome, we found that the genus *Candida* was significantly different in abundance between treatment groups.

From our results, our data presented a scenario when administering slightly different treatments resulted in similar abundances. Both treatment groups III (Meropenem/Vancomycin + Infloran) and VI (Meropenem/Vancomycin) exhibited similar family abundances. In addition, treatment groups III and VI presented taxa, *Enterobacteriaceae*, *Heliocobacteraceae* and *Lactobacillaceae*, that were absent in other treatment groups. Furthermore, although group VI did not receive Infloran^®^, the data in both groups showed an increase in *Lactobacillaceae* at the first time point. The lack of detectability of these highlighted family abundances at the second time would suggest that administering antibiotics may temporarily influence on the gut, and produce no long lasting effects. In contrast, our results also presented a case when administering probiotics, resulted in slightly different compositions. Treatment groups I (Ampicillin/Gentamicin + Infloran) and IV (Ampicillin/Gentamicin) were dominated by *Bacteroidaceae* in both time periods. Group I presented a development of *Heliocobacteraceae* at the second time point, however, group IV presented a noticeable abundance of *Prevotellaceae* at both time points. From this scenario, the data would suggest that administering Ampicillin/Gentamicin may produce lasting effects of the family *Prevotellaceae*. Furthermore, administering probiotics may have some association with the development of non beneficial/ disease contributing taxa. Finally, our data presented a scenario where administering antibiotics only resulted in taxa abundances that closely aligned with the no treatment group. Treatment groups IV (Ampicillin/Gentamicin) and VII (PBS oral) were both consistently comprised of *Bacteroidaceae* and *Heliocobacteraceae*. In other words, although both treatment groups received distinct treatments, the mice developed nearly identical gut compositions. This finding would suggest that administering Ampicillin/Gentamicin results in gut compositions similar to those in non treatment groups.

In alignment with other studies, we saw that in the Meropenem/Vancomycin groups, there was an increase in the phylum *Proteobacteria* and *Firmicutes* [43, 49]. Our results are concordant with another study such that *Lactobacillus* had a higher relative abundance only at the first time point and in the antibiotic with probiotics treatment group [81]. Our results differ from other studies such that we did not see any drastic decrease in taxa after the use of antibiotics or any shift in abundance after using probiotics [30, 40, 61, 82] In addition, we did not see an increase in ARGs in the antibiotics groups. We identified one antibiotic reisistance gene, Bla ACT-5, in group VI (Meropenem/Vancomycin) and only at the first time point. This result would strongly suggest that administering Meropenem/Vancomycin may influence the prevention of the ARG Bla ACT-5.

### Limitations and strengths

Our study differs from similar studies such that it included both preterm infants and neonatal mice, both amplicon (16S and ITS1) and WGS datasets and we performed analyses on the microbiome, resistome and mycobiome. By including both human and mice subjects, we were able to compare the same combination of treatments and potentially predict how long term human gut composition would develop. By acquiring both types of sequencing datasets, we were able to analyze the gut compositions on low and high resolutions. During the study period, we faced some minor challenges and would suggest some precautions for similar future research: Successful sample collecting and sequencing had been performed prior to this study, however, the low amount of bacterial and fungal DNA in both the preterm infant and neonatal mice samples suggest that there may be some form of contamination in our data. We speculate that the separation of fecal substance from the diapers and cages was too little and therefore, substances other than biomass may also have been sequenced. Furthermore, the specific pathogen free conditions, particularly the laboratory and neonatal intensive care unit, may have significantly reduced bacterial load. Even though extensive pretests were conducted, the storage and extraction methods may not have been optimal to retrieve high DNA volumes. In addition, the observational period of our study was potentially too short. Other similar studies had longer trial times, ranging from 3 months to two years. Studies have shown that lasting effects may need longer time to develop, which therefore, call for longer observational times. In future works, it may be beneficial to conducts longer trials in order to detect consistent and permanent effects [56, 87]. Direct comparison between microbiome samples and mycobiome samples was limited as two different pipelines existed in the dedicated laboratories for microbiome and mycobiome analyses. These pipelines comprised of two different DNA-extraction methods. Some studies have suggested and shown evidence that the gut microbiome and mycobiome do interact with each other and these interactions have influences on health, immunity and disease outcomes [10, 57, 71]. For this reason, it would be greatly beneficial for future studies to look into correlations and interactions between the gut bacterial and fungal communities. Further, due to limited amount of faeces and DNA not all samples could be used for both pipelines. Of course, having more samples would allow for stronger support on the inferences made. The probiotic, Infloran^®^, was not sequenced, thus direct comparison of bacterial sequences deriving from probiotics was not possible. However, the focus of our study was to compare preterm infants and mouse pups exposed to probiotics as used in clinical routine to those unexposed. In most gut composition clinical studies, it is of common practice to sequence and analyze stool samples. Emerging studies show that collecting stool samples may not be the most optimal way to observe the microbiome and resistome and suggest that direct sampling, such as endoscopy samples, would be preferable [25, 60, 92]. While analyzing mice provides a form of standardization between different studies, mice models have their own limitations [13, 42]. For example, a study observed that mice obtained from four different vendors displayed predominantly similar microbiome compositions. However, notable variations in gut compositions were also detected, more specifically, certain mice exhibited unique taxa specific to each respective vendor [58]. Moreover, the probiotic, Infloran^®^, is not an appropriate medical supplement to enhance or improve the gut composition of mice. Despite the limitations, mice have already and are still included in the experimentation of infant antibiotics and probiotics [43, 60, 65, 91].

## Conclusions

The objective of this clinical study aimed to analyze the impact of antibiotics and probiotics on three components of the gut composition, namely the microbiome, resistome and mycobiome, in both preterm infants and neonatal mice. The data did not show stable and significant effects of antimicrobial therapy in preterm infants nor neonatal mice on the general abundances of bacterial communities in the gut. We found wide heterogeneity in the microbiome, resistome, and mycobiome composition between individuals at different time points. In addition, twenty-seven antibiotic resistant genes were found when analyzing the resistome. The mycobiome, however, did differ significantly between treatment groups, a result underlining the need for further mycobiome analyses.

Our work on administering medical interventions to preterm infants produced transient effects. The outcomes produced provide positive motivation to continue this research with a focus on environmental and genetic factors to further understand how antibiotics, probiotics and non medical factors influence the preterm infant gut microbiome, resistome and mycobiome.

### Supplementary Information

**Additional file 1: Table S1. **Full allocation of medical interventions among the 58 preterm infants included in this study.

**Additional file 2: Table S2.** Full distribution of medical interventions among the 48 neonatal mice included in this study.

**Additional file 3: Table S3.** Percentage of preterm infant samples mapping to ARG Genes with a sequence similarity search cutoff of 97%.

**Additional file 4: Table S4.** Full distribution of multiple births. One set of triplets and 7 pairs of twins. All multiple births, with the exceptions of the set of triplets and one pair of twins, were in the same treatment group. Of the set of triplets, infant 19 received antibiotics with the addition of probiotics and infants 20 and 21 received only probiotics. Infant 1 and infant 2 are a pair of twins, but did not receive the same treatment, one received only probiotics and one received no treatment respectively. Infant twins 106 and 107 were in the same treatment group and both received Ampicillin, Gentamicin, and Vancomycin, however, infant 107 received an additional antibiotic, Unacid.

## Data Availability

Raw sequencing data from ITS1 gene sequencing has been submitted to the NCBI Sequence Read Archive under the BioProject (BioProjectID: PRJNA909072,http://www.ncbi.nlm.nih.gov/bioproject/909072). Raw sequencing WGS-data has been submitted to: https://www.ebi.ac.uk/ena/browser/view/PRJEB74138.

## Abbreviations

NICU: Neonatal intensive care units
NEC: Necrotizing enterocolitis
ARG: Antibiotic resistance genes
rRNA: Ribosomal ribonucleic acid
ITS: Internal transcribed spacer
WGS: Whole genome sequencing
ANCOM: Analysis of compositions of microbiomes
CARD: Comprehensive Antibiotic Resistance Database
ARG-ANNOT: Antibiotic Resistance Gene-Annotation
OTU: Operational taxonomic units
PERMANOVA: Permutational multivariate analysis of variance
EOS: Early onset sepsis
LOS: Late onset sepsis
Bla: Beta lactamase
Sul: Sulfonamide
Tet: Tetracycline
GLMM: Generalized linear mixed model
PBS: Phosphate buffered saline
