# Supplementary material for: The gut microbiome, resistome, and mycobiome in preterm newborn infants and mouse pups: lack of lasting effects by antimicrobial therapy or probiotic prophylaxis
Source: Gut Pathog. 2024 May 12;16:27. doi: 10.1186/s13099-024-00616-w (PMC11089716; doi:10.1186/s13099-024-00616-w)
Supplement: Supplementary file 1 — Additional file 1: Table S1. Full allocation of medical interventions among the 58 preterm infants included in this study. [file 13099_2024_616_MOESM1_ESM.pdf]

	No antibiotics	Antibiotics	Ampicillin/ Gentamicin	Gentamicin/ Meropenem	Unacid/ Vancomycin	Ampicillin/ Clindamycin	Unacid/ Gentamicin/ Vancomycin	Unacid/ Gentamicin/ Clarithromycin	Unacid/ Gentamicin/ Flucloxacin	Ampicillin/ Vancomycin/ Meropenem	Sum
No probiotics	13	1	1	0	0	0	0	0	0	0	14
Probiotics	21	23	13	3	2	1	1	1	1	1	44
Sum	34	24	14	3	2	1	1	1	1	1	58

Table S1. Full allocation of medical interventions among the 58 preterm infants included in this study
