# Supplementary material for: The gut microbiome, resistome, and mycobiome in preterm newborn infants and mouse pups: lack of lasting effects by antimicrobial therapy or probiotic prophylaxis
Source: Gut Pathog. 2024 May 12;16:27. doi: 10.1186/s13099-024-00616-w (PMC11089716; doi:10.1186/s13099-024-00616-w)
Supplement: Supplementary file 2 — Additional file 2: Table S2. Full distribution of medical interventions among the 48 neonatal mice included in this study. [file 13099_2024_616_MOESM2_ESM.pdf]

ID number	Treatment group	Medical intervention	Number of samples for microbiome	Number of samples for mycobiome
I	Antibiotics + probiotics	Ampicillin/Gentamicin + Infloran	14	13
II	Only probiotics	Infloran	9	7
III	Antibiotics + probiotics	Meropenem/Vancomycin+ Infloran	12	11
IV	Only antibiotics	Ampicillin/Gentamicin	10	8
VI	Only antibiotics	Meropenem /Vancomycin	11	9
VII	No treatment	None (PBS oral)	14	13

Table S2. Full distribution of medical interventions among the 48 neonatal mice included in this study.
