# Supplementary material for: The gut microbiome, resistome, and mycobiome in preterm newborn infants and mouse pups: lack of lasting effects by antimicrobial therapy or probiotic prophylaxis
Source: Gut Pathog. 2024 May 12;16:27. doi: 10.1186/s13099-024-00616-w (PMC11089716; doi:10.1186/s13099-024-00616-w)
Supplement: Supplementary file 3 — Additional file 3: Table S3. Percentage of preterm infant samples mapping to ARG Genes with a sequence similarity search cutoff of 97%. [file 13099_2024_616_MOESM3_ESM.pdf]

Treatment group	Antibiotics + probiotics			No treatment			Only probiotics			Only antibiotics		
**Day of life**	**01-08**	**09-17**	**18-69**	**01-08**	**09-17**	**18-69**	**01-08**	**09-17**	**18-69**	**01-08**	**09-17**	**18-69**
**AGly**	4.17	8.33	8.33	-	-	12.5	-	4.35	7.69	-	-	-
**Bla**	12.5	25	41.67	7.14	13.33	-	-	-	-	-	-	-
**Flq**	-	4.17	4.17	-	-	-	-	-	-	-	-	-
**MLS**	4.17	4.17	4.17	-	-	-	4.17	8.7	3.85	-	-	-
**Sul**	-	4.17	4.17	-	-	12.5	-	-	26.92	-	-	-
**Tet**	-	-	-	-	-	-	-	-	23.08	-	-	-
**Tmt**	4.17	-	-	-	-	-	4.17	30.43	-	-	-	-

Table S3 Using the ARG-ANNOT database, we observed what percentage of our preterm infant samples mapped 97% to at least one gene in the ARG class.
