# Supplementary material for: The gut microbiome, resistome, and mycobiome in preterm newborn infants and mouse pups: lack of lasting effects by antimicrobial therapy or probiotic prophylaxis
Source: Gut Pathog. 2024 May 12;16:27. doi: 10.1186/s13099-024-00616-w (PMC11089716; doi:10.1186/s13099-024-00616-w)
Supplement: Supplementary file 4 — Additional file 4: Table S4. Full distribution of multiple births. One set of triplets and 7 pairs of twins. All multiple births, with the exceptions of the set of triplets and one pair of twins, were in the same treatment group. Of the set of triplets, infant 19 received antibiotics with the addition of probiotics and infants 20 and 21 received only probiotics. Infant 1 and infant 2 are a pair of twins, but did not receive the same treatment, one received only probiotics and one received no treatment respectively. Infant twins 106 and 107 were in the same treatment group and both received Ampicillin, Gentamicin, and Vancomycin, however, infant 107 received an additional antibiotic, Unacid. [file 13099_2024_616_MOESM4_ESM.pdf]

	# of Sets	Antibiotics	Antibiotics + probiotics	Probiotics	None
Triplets	1		19	20,21	
Twins	7		(9,10)<br>(106,107)	(22,23)<br>(101,26)<br>(108,109)<br>(121, 122)<br>1	2

Table S4. Full distribution of multiple births. One set of triplets and 7 pairs of twins. All multiple births, with the exceptions of the set of triplets and one pair of twins, were in the same treatment group. Of the set of triplets, infant 19 received antibiotics with the addition of probiotics and infants 20 and 21 received only probiotics. Infant 1 and infant 2 are a pair of twins, but did not receive the same treatment, one received only probiotics and one received no treatment respectively. Infant twins 106 and 107 were in the same treatment group and both received Ampicillin, Gentamicin, and Vancomycin, however, infant 107 received an additional antibiotic, Unacid.
